# UBE2V2 promotes metastasis by regulating EMT and predicts a poor prognosis in lung adenocarcinoma

**DOI:** 10.1002/cam4.6566

**Published:** 2023-09-27

**Authors:** Zheng Yang, Gujie Wu, Jianmei Zhao, Guanglin Shi, Juan Zhou, Xiaoyu Zhou

**Affiliations:** ^1^ Department of Respiratory Medicine Affiliated Hospital of Nantong University Nantong China; ^2^ Department of Thoracic Surgery Zhongshan Hospital Fudan University shanghai China; ^3^ Department of Pediatrics Affiliated Hospital of Nantong University Nantong China; ^4^ Department of respiratory medicine The sixth people's hospital of Nantong Nantong China

**Keywords:** apoptosis and proliferation, bioinformatic, EMT, LUAD, UBE2V2

## Abstract

**Purpose:**

As a member of the ubiquitin‐conjugating enzyme (E2) family, UBE2V2 demonstrates significant tumorigenicity in many cancers. However, the relationship between UBE2V2 expression and the morbidity of lung adenocarcinoma (LUAD) is still unknown.

**Methods:**

We detected the mRNA and protein expression of UBE2V2 and analyzed its relationship with clinical parameters as well as survival prognosis based on bioinformatic and immunohistochemistry (IHC) in LUAD. The signaling pathway of UBE2V2 in the development of LUAD was obtained by GSEA. The TIMER database was used to investigate the association between UBE2V2 expression and the level of infiltration of different immune cells. Finally, we explored the effects of UBE2V2 knockdown on the proliferation, apoptosis, and migration of LUAD cells.

**Results:**

The results showed that UBE2V2 was a potential oncogene and might be considered an independent prognostic molecule for LUAD patients based on TCGA prediction (HR: 1.497 *p* = 0.012) and IHC (HR:1.864 *p* = 0.044). IHC showed that UBE2V2 was related to the following clinicopathological factors: gender (*p* = 0.043), stage (*p* = 0.042), and lymph node metastasis (*p* = 0.002). Finally, knockdown of UBE2V2 reduced the migration of LUAD cells by regulating EMT‐related proteins. Knockdown of UBE2V2 induced LUAD cells to arrest in the G1 phase. Knockdown of UBE2V2 increased LUAD cell apoptosis and decreased proliferation, which might be related to the downregulation of PCNA and upregulation of P53 and ƳH2AX expression. Interestingly, UBE2V2 is negatively correlated with B cells, CD4+ T cells, macrophages, and dendritic cells.

**Conclusion:**

UBE2V2 may be a valuable therapeutic target for lung cancer.

## 1 INTRODUCTION

Lung cancer poses a threat to male mortality, which is second to breast cancer for women.^1^ Lung cancer is composed of two types: non‐small cell lung cancer (NSCLC) and small‐cell lung cancer (SCLC). Among lung cancers, NSCLC accounts for approximately 80% and mainly constitutes lung adenocarcinoma (LUAD) and lung squamous cell carcinoma (LUSC).^2^ LUAD is the most common histological subtype based on the clinicopathological diagnosis.^3^ Most LUAD patients are diagnosed as locally advanced or metastatic, and the early metastasis of lung cancer often results in a higher degree of malignancy and poor treatment effect.^4^ In recent years, despite having more diagnostic technologies and novel treatment methods, a large number of LUAD patients are still diagnosed at an advanced stage, so their 5‐year overall survival (OS) remains low.^5^ Hence, it is essential to discover an effective molecular biomarker to increase the long‐term survival prognosis of LUAD patients, which can be used for the early diagnosis and treatment of LUAD.

Recently, many studies have found that the ubiquitin‐protease system (UPS) is related to the occurrence of many malignant tumors. Ubiquitylation is mediated via a series of enzymes. First, ubiquitin (Ub)‐activating enzymes (E1) hydrolyze ATP to form high‐energy thioesters between the COOH terminus of Ub and the cysteine of E1. Activated ubiquitin is delivered to ubiquitin‐conjugating enzymes (E2) and transferred diametrically to substrate proteins or ubiquitin‐ligase enzymes (E3).^6^ Ubiquitin‐conjugating E2 enzyme variants (UBE2V2) belong to the ubiquitin family, which contains two family members, UBE2V1 and UBE2V2.^7^, ^8^ UBE2V2 (otherwise called hMMS2; DDVIT1; EDAF‐1) is essential for genome maintenance in the nucleus through DNA damage repair pathways.^9^ Overexpression of UBE2V2 induces the occurrence of many types of tumors.^10^, ^11^ UBE2V2 was highly expressed in malignant melanoma, and knocking down it reduced melanoma cell proliferation and subcutaneous tumor growth.^12^ UBE2V2 was highly expressed in prostate cancer, and it has been confirmed that miR‐499a inhibits the proliferation of human prostate cancer cells by targeting UBE2V2.^13^ In lung adenocarcinoma, overexpression of UBE2V2 is positively correlated with PD‐L1 mRNA level, T classification, and poor survival rate in LUAD patients based on TCGA and IHC.^13^ However, the mechanism of UBE2V2 development in malignant progression of LUAD cells has not been investigated in vitro.

In this report, we jointly analyzed the mRNA and protein levels of UBE2V2 in LUAD and normal samples in light of the prediction of bioinformatics, IHC, and Western blotting (WB). Meanwhile, we analyzed the relationship between UBE2V2 and clinicopathological parameters, and survival prognosis. Furthermore, we studied whether knockdown of UBE2V2 affected the invasion, migration, proliferation, and apoptosis of LUAD cells in vitro. We studied the relationship between UBE2V2 and EMT‐related proteins through WB. Interestingly, we also found that UBE2V2 regulated the level of immune cell infiltration in LUAD.

## 2 METHODS

### 2.1 Collection of RNA‐sequencing data based on the TCGA database

LUAD sample mRNA (551 samples in total, including 54 normal samples) and matched clinical characteristic parameters were acquired from TCGA (https://portal.gdc.cancer.gov/). TCGA uses large‐scale high‐throughput genome sequencing analysis technology to assist in better understanding tumors, thereby improving cancer prevention, diagnosis, and treatment capabilities. The Illumina HiSeq RNA‐Seq platform provided all LUAD sequencing data and was used to analyze the role of UBE2V2 in LUAD. After removing samples with incomplete survival information and those lost to follow‐up from TCGA‐LUAD, Cox regression was used to analyze 439 samples with complete clinical information. Co expression analysis was used to predict the possible upstream and downstream genes of UBE2V2. Based on TCGA, Strawberry Perl and R (3.5.2) software were used for bioinformatics analysis.

### 2.2 PrognoScan database analysis

The PrognoScan database (http://www.abren.net/PrognoScan/) was used to examine the relationship between UBE2V2 expression and survival in LUAD.^14^ PrognoScan is the most comprehensive database for survival analysis information, mainly sourced from GEO. The cutoff was changed to a Cox *p* value of less than 0.05.

### 2.3 Gene set enrichment analysis of UBE2V2

GSEA is a calculation used to explain that a gene shows a marked significant difference between the high and low groups. We carried out GSEA analysis on the normalized RNA‐Seq data obtained from TCGA.^15^ We uploaded the generated dataset and phenotype label file to GSEA software. After the phenotype was marked as UBE2V2 high and UBE2V2 low, the KEGG pathway of UBE2V2 in GSEA was acquired. *p* < 0.05 and FDR <0.05 were regarded as enrichment.

### 2.4 Correlation between UBE2V2 and immune infiltrating cells

The “Gene” module of TIMER was used to investigate the association between UBE2V2 and a variety of immune cells (CD4^+^ T cells, dendritic cells, B cells, CD4^+^ T cells, B cells, neutrophils, and macrophages) and tumor purity in LUAD. TIMER (https://cistrome.shinyapps.io/timer/) systematically analyzed the immune infiltration of various cancers.

### 2.5 Tissue samples of patients

Between 2013 and 2020, specimens of 91 LUAD patients who had not received any radiotherapy or chemotherapy before undergoing surgery at Nantong University Affiliated Hospital were collected for IHC analysis. Twelve pairs of tissues were randomly selected from 91 LUAD samples for WB. The patients participating in this study were informed that their tissues and related clinical information would contribute to scientific research and signed an informed consent form. The Ethics Committee of Nantong University Affiliated Hospital agreed to the application of these specimens and data in scientific research.

### 2.6 Immunohistochemistry

We fixed the fresh tissue samples with 10% formalin before embedding. Then, these paraffin‐embedded tissue samples were cut into four‐micron‐thick tissue slices. After 6 h of baking in a 60°C incubator, the tissue sections were immersed in ethanol at different concentrations for dewaxing. To block the activity of endogenous peroxidase, 3% hydrogen peroxide (H_2_O_2_) was dripped onto the surface of the tissue sections at 121°C. The tissue sections were incubated with anti‐UBE2V2 polyclonal antibody (dilution 1:100, cat. no. D624139‐0001; Sangon Biotech) at 25°C for 2 h. PBS solution was used to wash the tissue sections three times, each for 10 min. The secondary antibody matched with the anti‐UBE2V2 polyclonal antibody was dripped onto the surface of the tissue section at 25°C for 1 h. The DAB solution was dripped onto the surface of the tissue section to catalyze the formation of a water‐insoluble brown–yellow product for visualization. After being repeatedly washed with water, the tissue sections were stained with hematoxylin, and then the sections were covered with neutral resin and sealed with cover glasses. The staining results of the tissue sections were evaluated by three experienced pathologists. The proportion of stained cells in each tissue section was scored based on the following system: 1 (1%–30%), 2 (31%–50%), 3 (51%–70%), or 4 (71%–100%). The staining intensity of the tissue sections was scored based on the following criteria: 0 (no staining), 1 (light yellow), 2 (deep yellow), and 3 (brown particles). According to the product of the two scores, UBE2V2 expression was divided into high‐ and low‐score groups: the low‐score group was from 1 to 4, and the high score group was from 6 to 12.^16^

### 2.7 RNA extraction and quantitative RT‐PCR

Twenty‐two pairs of fresh LUAD from IHC were used to extract RNA. Total RNA was isolated from LUAD samples and four cells using TRIzol reagent (Invitrogen, Carlsbad, CA, USA). Reverse transcription of total RNA into complementary DNA (cDNA) using PrimeScript RT Reagent Kit (TaKaRa, Japan). The primers for RT‐PCR, UBE2V2 forward: 5′‐CACGGTCTATTCCATCCACATC‐3′, UBE2V2 reverse: 5′‐GGTTCGCAAAACAATCGGCT‐‐3′, GAPDH forward 5′‐AACAGCCTCAAGATCATC ‐3′, reverse 5′‐CACGATACCAAAGTTGTC ‐3′, were purchased from Sangon (Shanghai, China). RT‐qPCR was performed using the ABI 7500 FAST Real‐Time PCR System (Applied Biosystems, Carlsbad, CA, USA) and SYBR Green Master Mix (Vazyme, Nanjing, China). UBE2V2 expression was normalized to GAPDH using the 2^−ΔΔCt^ relative quantification method.

### 2.8 Western blotting

Lysis buffer was added to the crushed fresh LUAD tissue and cells and cultured for 48 h to collect protein. After centrifugation at 12000 rpm for one‐quarter of an hour, the protein‐containing supernatant was transferred to an enzyme‐free centrifuge tube. A BioRad protein assay was used to quantify the protein concentration (BioRad Laboratories, Inc.). At a voltage of 120 V, SDS–PAGE gels were used to separate proteins from different samples. Then, the protein on the gel was transferred to a PVDF (polyvinylidene fluoride) microporous membrane at an electric current of 200 mA. The PVDF membrane containing protein was soaked in 5% skim milk at 25°C for 2 h. The diluted primary antibody was added to the blocked PVDF membrane and incubated at 4°C for 12 h. The membrane was washed with TBST 3 times for 20 min each time. The HRP‐conjugated secondary antibody (dilution 1:10000, cat. no. A0208; Beyotime Institute of Biotechnology) was added to the PVDF membrane containing the protein and incubated for 2 h at 25°C.Similarly, TBST was used to wash the PVDF membrane 3 times for 20 min each time. The primary antibody anti‐UBE2V2 (D624139‐0001) was purchased from Sangon Biotech. Anti‐GAPHD (10494‐1‐AP), anti‐MMP2 (10373‐2‐AP), antivimentin (10366‐1‐AP), anti‐E‐cadherin (20874‐1‐AP), anti‐PCNA (10205‐2‐AP), and anti‐N‐cadherin (22018‐1‐AP) were acquired from Proteintech. Finally, the protein bands separated by gel electrophoresis were recognized by an ECL detection system (Pierce, Rockford, IL, USA).

### 2.9 Cell culture and transfection

In the present study, BEAS‐2B, H1299, A549, SPCA1, and H1650 cell lines were acquired from the Shanghai Institute of Cell Biology Academia, and cell lines have been identified by STR. These cells were cultured in RPMI 1640 medium containing 10% fetal bovine serum (FBS). Cancer cells in logarithmic growth were generally used in experiments from the third to sixth generation. For UBE2V2 silencing, three different UBE2V2‐shRNA lentiviruses and one Con‐shRNA lentivirus were designed via GeneChem Technologies: primer sequence (5′‐3′) of shRNA‐1 GCCCGGAGCATACCAGTGTTA; primer sequence (5′‐3′) of shRNA‐2 CAAGGTGGACAGGCATGATTA; primer sequence (5′‐3′) of shRNA‐3 GTCTTAAATCAACAACCTTCT; and primer sequence (5′‐3′) of Con077 (shCon) TTCTCCGAACGTGTCACGT. A549 and SPCA1 cells were selected for lentiviral transfection. After 24 h, the cell culture medium was replaced, and stable UBE2V2‐shRNA‐expressing cells were obtained after selection with puromycin. In the above experiment, we strictly followed the manufacturer's reference guide.

### 2.10 Cell migration assays

The collected 2 × 10^4^ cells were transferred into a Transwell chamber in a 24‐well plate. Five hundred microlitres of medium with FBS was placed into the lower chamber of the 24‐well plate to facilitate cell migration. After 48 h, methanol was used to fix the cells that migrated to the lower chamber of the 24‐well plate for 20 min, and 0.1% crystal violet staining was used for cell staining for half an hour. A microscope (Olympus, Japan) with a 40× objective lens was used to take photographs and record the migrated cells (4 fields/chamber × triplicate).

### 2.11 Cell cycle analysis

After collecting the cells in logarithmic growth in 6‐well plates, precooled PBS was used to wash the cells twice (3 mL, suspended, and centrifuged), and then the collected cells were fixed in precooled 75% absolute ethanol at 4°C overnight. The cells were stained with propidium iodide (PI) solution (50 μg/mL PI, 100 μg/mL RNase A, and 0.2% Triton X‐100; Sigma) for half an hour at 4°C in the dark. The processed cells were identified by Attune NxT flow cytometry to determine whether they were in the G1, S, or G2/M phases.

### 2.12 Apoptosis analysis

Adherent shCon and shUBE2V2 cells (1 × 10^6^/mL) were trypsinized and collected in a 10 mL centrifuge tube. These cells were rinsed twice with precooled PBS. Immediately afterward, the cells were centrifuged at 4°C for 5 min at 2000 g. Then, the cells were incubated with 5 μL Annexin V, 5 μL PI together with a fluorescent dye (Meilunbio^R^, China) in the dark at 25°C for 15 min. Finally, flow cytometry (FACSCalibur, Becton‐Dickinson, USA) was used to detect cell apoptosis.

### 2.13 Colony formation assay

Cells in logarithmic division stage were transferred into six‐well plates (800 cells/well). After 10 days of cultivation, the cloned cells were immersed in methanol for fixation for 30 min and stained with Giemsa solution for a quarter of an hour. The number of colonies was recorded by fluorescence microscopy (Olympus). The colony‐forming assay was repeated three times in duplicate.

### 2.14 EDU assay

Cell‐Light EdU DNA Cell Proliferation Kit (RIBOBIO, Guangzhou, China) was used to analyze cell proliferation ability. Cells (2 × 10^4^) in logarithmic growth phase were seeded into 6‐well plates and cultured for 48 h. Then 50 μmoL/L EdU was added to the six‐well plate (2 h), and then cells were fixed with 4% paraformaldehyde solution. 500 μL 1× Apollo® was added to cells for staining reaction (30 min) and incubated with 0.5% Triton X‐100. Finally, the cells were resuspended in PBS and imaged under a fluorescence microscope.

### 2.15 Statistical analysis

We applied GraphPad Prism 8.0 and SPSS 24.0 software to perform statistical analysis. We applied an unpaired Student's *t*‐test to the comparison of two groups. Multiple comparisons were analyzed using ANOVA with posttest. We applied the Kaplan–Meier method to predict the survival rate of patients. *p* < 0.05 was regarded as a significant difference. All experiments in this study were repeated three times.

## 3 RESULTS

### 3.1 UBE2V2 expression was upregulated in LUAD tissues compared to normal samples

**FIGURE 1:**
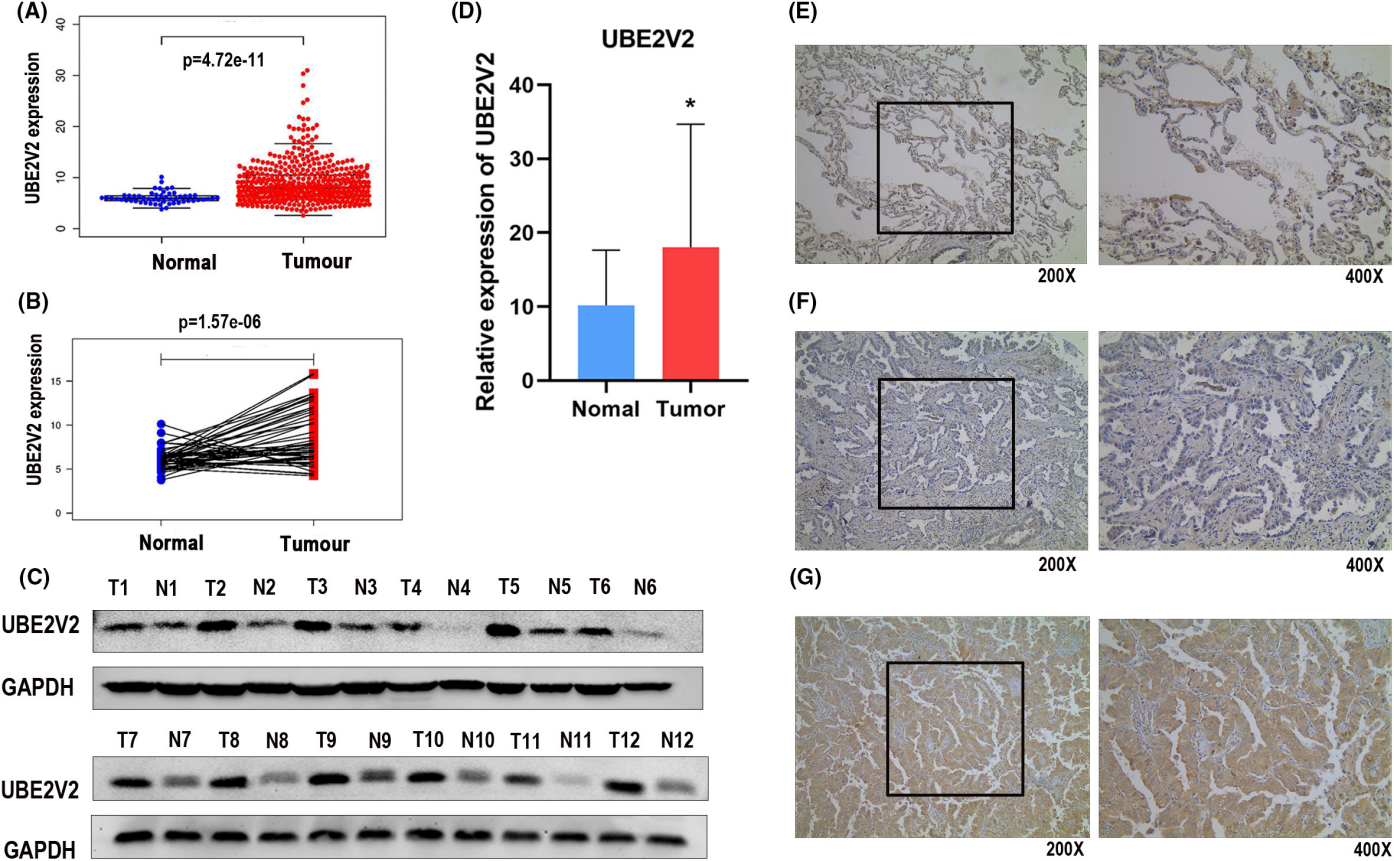
The mRNA and protein expression of UBE2V2 in LUAD. (A) The mRNA expression of UBE2V2 between 497 LUAD tissues and 54 normal samples from TCGA (red dots represent tumor samples, and the blue dots represent normal samples). (B) The mRNA expression of UBE2V2 in 54 LUAD samples and its matched vicinal samples (red dots represent tumor samples and the blue dots represent normal samples). (C) Protein expression of UBE2V2 in LUAD (T) and adjacent normal tissues (N) measured by Western blotting. (D) The mRNA levels of UBE2V2 in the 22 pairs of fresh LUAD and normal adjacent tissues were measured by PCR (red representstumor samples and the blue represents normal samples). (E) Normal lung tissue with negative expression of UBE2V2; (F) LUAD tissue with negative expression of UBE2V2; (G) LUAD tissue with positive expression of UBE2V2. The data (mean ± standard deviation) between two groups were compared using unpaired *t*‐test. *p < 0.05; (UBE2V2, ubiquitin‐conjugating E2 enzyme variants 2; TCGA, The Cancer Genome Atlas; LUAD, lung adenocarcinoma; PCR, polymerase chain reaction).

The 497 LUAD and 54 normal samples obtained from the TCGA network platform were analyzed by R (v 3.5.2). We compared the mRNA level of UBE2V2 between LUAD and adjacent normal samples. The results indicated that UBE2V2 mRNA expression in LUAD tissues was remarkably higher than that in normal tissues (*p* < 0.001) (Figure 1A, B). IHC showed that UBE2V2 expression was different in 91 LUAD samples (UBE2V2 was highly expressed in 50 LUAD samples, as shown in Table 1). IHC showed the staining intensity of UBE2V2 in normal lung tissues and LUAD tissues with different degrees of differentiation (Figure 1E: normal lung tissue with negative expression of UBE2V2; Figure 1F: LUAD tissue with negative expression of UBE2V2; Figure 1G: LUAD tissue with positive expression of UBE2V2). To further validate the results of the above bioinformatics prediction, WB and PCR were used to detect the protein and mRNA levels of UBE2V2 in fresh LUAD and normal adjacent tissues, respectively. The results revealed that UBE2V2 protein (Figure 1C) and mRNA expression (Figure 1D) was remarkably upregulated in LUAD. The above results indicated that UBE2V2 was a potential oncogene in LUAD that might promote the malignant development of LUAD patients.

**TABLE 1:** Association between UBE2V2 expression and pathological parameters of LUAD in the immunohistochemistry.

Clinical parameters	All cases	UBE2V2 expression		*p* Value
		Low	High	
Total	91	41	50	
Age				
≤65	58	28	30	0.413
>65	33	13	20	
Gender				
Male	59	22	37	**0.043**
Female	32	19	13	
Smoking				
Nonsmoker	71	33	38	0.607
Smoker	20	8	12	
Stage				
I	47	26	21	**0.042**
II + III + IV	44	15	29	
T classification				
T1 + T2	85	38	48	0.490
T3 + T4	6	3	2	
Distant metastasis status				
M0	87	39	48	0.613
M1	4	2	2	
Lymph node metastasis				
N0	51	31	20	**0.002**
N1	21	7	14	
N2	19	3	16	
Differentiated degree				
Low + Middle grade	88	39	49	0.444
High grade	3	2	1	

*Note*: Bold means *p* < 0.05 was considered to indicate a statistically significant difference.

### 3.2 UBE2V2 was associated with poor prognosis in LUAD patients

**FIGURE 2:**
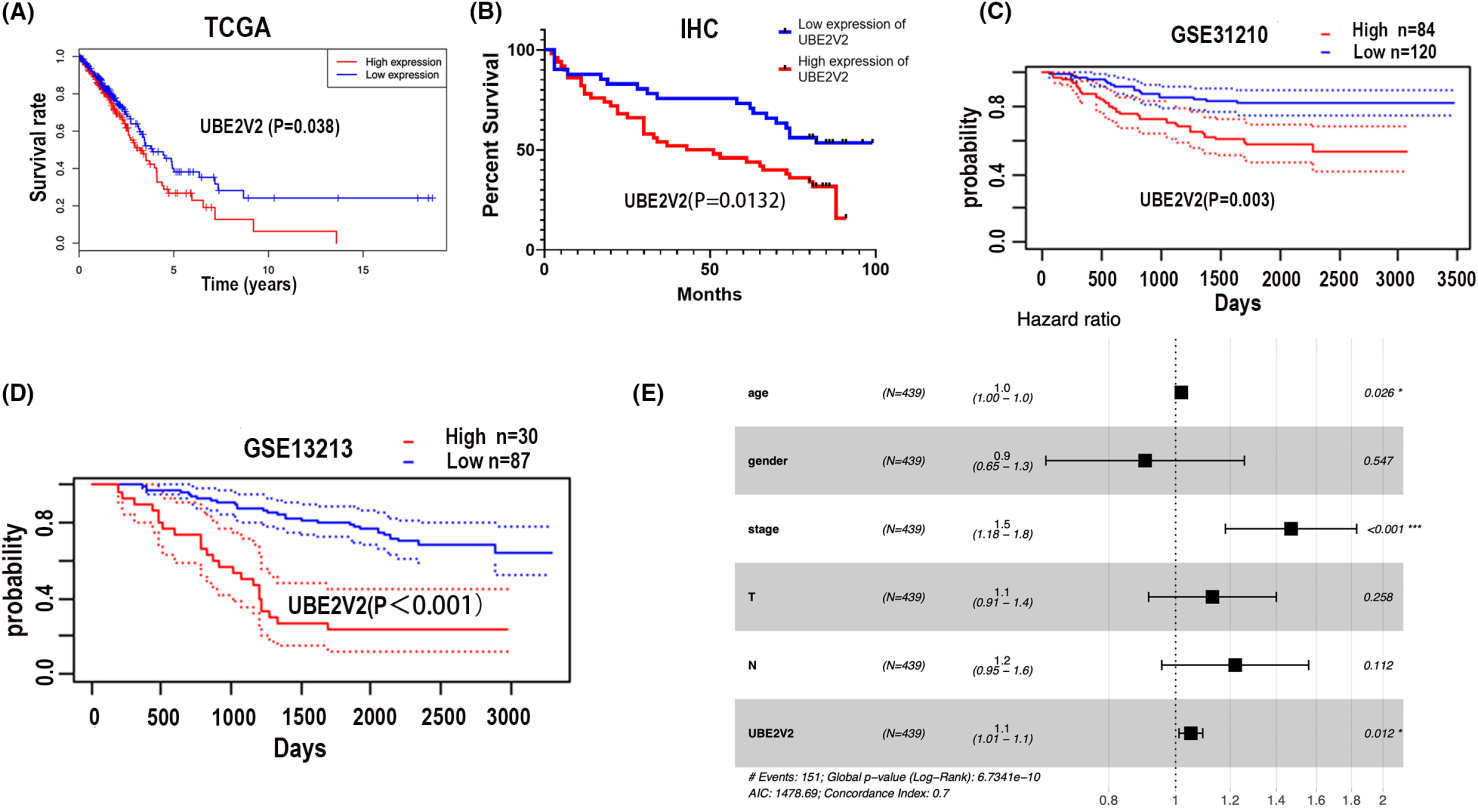
The expression of UBE2V2 was bound up with the overall survival in LUAD patients. (A) Survival curve of UBE2V2 expression based on TCGA database in LUAD patients (the red curve represents high expression of UBE2V2, and the blue curve represents low expression of UBE2V2). (B) Percent survival of UBE2V2 in 91 pairs LUAD tissues from IHC (the red curve represents high expression of UBE2V2, and the blue curve represents low expression of UBE2V2). (C and D) Overall survival was obtained through PrognoScan online database (the red curve represents high expression of UBE2V2, and the blue curve represents low expression of UBE2V2). (E) Multivariate COX analysis was calculated based on the clinicopathological parameters carried by 439 patients in the TCGA database. (UBE2V2, ubiquitin‐conjugating E2 enzyme variants 2; TCGA, The Cancer Genome Atlas; LUAD, lung adenocarcinoma; IHC, immunohistochemistry).

In TCGA, the “survival” package was used to draw survival curves of LUAD patients. The overall survival (OS) analysis illustrated that LUAD patients with high UBE2V2 expression had a worse prognosis than those with low UBE2V2 expression (*p* = 0.038) (Figure 2A). Two gene sets (GSE31210 and GSE13213) were selected based on the PrognoScan website. The results showed that high expression of UBE2V2 (GSE31210 *p* = 0.003 and GSE13213 *p* < 0.001) predicted poorer survival in lung adenocarcinoma patients (Figure 2C,D). Finally, we used the survival time information derived from 91 samples with IHC to draw a survival curve. The percent survival according to IHC analysis verified the above results: UBE2V2 may lead to poor survival prognosis in LUAD patients (*p* = 0.0132) (Figure 2B). Meanwhile, the univariate analysis based on TCGA forecasted that stage (HR: 1.65; 95% CI: 1.42–1.92; *p* < 0.01), T classification (HR: 1.53; 95% CI: 1.25–1.87; *p* < 0.01), N classification (HR: 1.69; 95% CI: 1.41–2.03; *p* < 0.01), and UBE2V2 expression (HR: 1.46; 95% CI: 1.07–2; *p* = 0.014) were remarkably related to overall survival (Table 2). Multivariate analysis, which was depicted as a forest boxplot, forecasted that UBE2V2 expression (*p* = 0.012) (Figure 2E and Table 2) was an independent prognostic factor for LUAD based on bioinformatics. The same results were verified by IHC analysis. In the IHC analysis, univariate analysis determined that the expression of UBE2V2 (HR: 2.002, 95% CI: 1.139–3.517, *p* = 0.016) and N classification (HR: 1.736, 95% CI: 1.016–2.964, *p* = 0.043), respectively, affected the survival of LUAD patients according to all variables (Table 3). Multivariate analysis indicated that UBE2V2 (HR: 1.864, 95% CI: 1.016–3.420, *p* = 0.044) could be considered an independent prognostic factor in LUAD (Table 3). In summary, UBE2V2 might be predicted as an adverse molecular marker for the malignant progression of LUAD based on the results of bioinformatics and IHC.

**TABLE 2:** Univariate and multivariate analysis of the relationship between UBE2V2 and overall survival in the TCGA.

Parameter	Univariate analysis			Multivariate analysis		
	HR	95% CI	*p* Value	HR	95% CI	*p* Value
Age	1.012	0.995–1.029	0.145	1.018	1.002–1.036	**0.025**
Gender	1.084	0.787–1.493	0.619	0.903	0.649–1.258	0.054
Stage	1.655	1.426–1.921	**3.07** ^**−11**^	1.474	1.184–1.835	**0.0005**
T	1.535	1.259–1.872	**2.24** ^**−05**^	1.122	0.905–1.391	0.292
M	─	─	─	─	─	─
N	1.699	1.419–2.033	**7.17** ^**−09**^	1.213	0.949–1.551	0.121
UBE2V2	1.469	1.078–2.002	**0.014**	1.497	1.073–2.089	**0.012**

*Note*: Bold means *p* < 0.05 was considered to indicate a statistically significant difference.

Abbreviations: CI, confidence interval; HR, hazard ratio.

**TABLE 3:** Univariate and multivariate analysis of the relationship between UBE2V2 and overall survival in the immunohistochemistry.

Parameter	Univariate analysis			Multivariate analysis		
	HR	95% CI	*p* Value	HR	95% CI	*p* Value
UBE2V2						
High vs. low	2.002	1.139–3.517	**0.016**	1.864	1.016–3.420	**0.044**
Age (years)						
≤65 vs. >65	1.345	0.786–2.303	0.279			
Gender						
Male vs. female	0.678	0.378–1.128	0.194			
T classification						
T1 vs. T2 vs. T3 + T4	1.157	0.831–1.611	0.388			
N classification						
Yes vs. no	1.736	1.016–2.964	**0.043**	1.117	0.795–1.569	0.052
M classification						
Yes vs. no	0.621	0.151–2.551	0.508			
Different degree						
low +middle vs. high	0.664	0.247–1.788	0.418			
TNM stage						
I vs. II vs. III + IV	1.127	0.634–2.299	0.411			

*Note*: Bold means *p* < 0.05 was considered to indicate a statistically significant difference. UBE2V2 and lymph node metastasis were included in multivariate analysis.

Abbreviations: CI, confidence interval; HR, hazard ratio.

### 3.3 Correlations between the expression of UBE2V2 and clinical parameters

**FIGURE 3:**
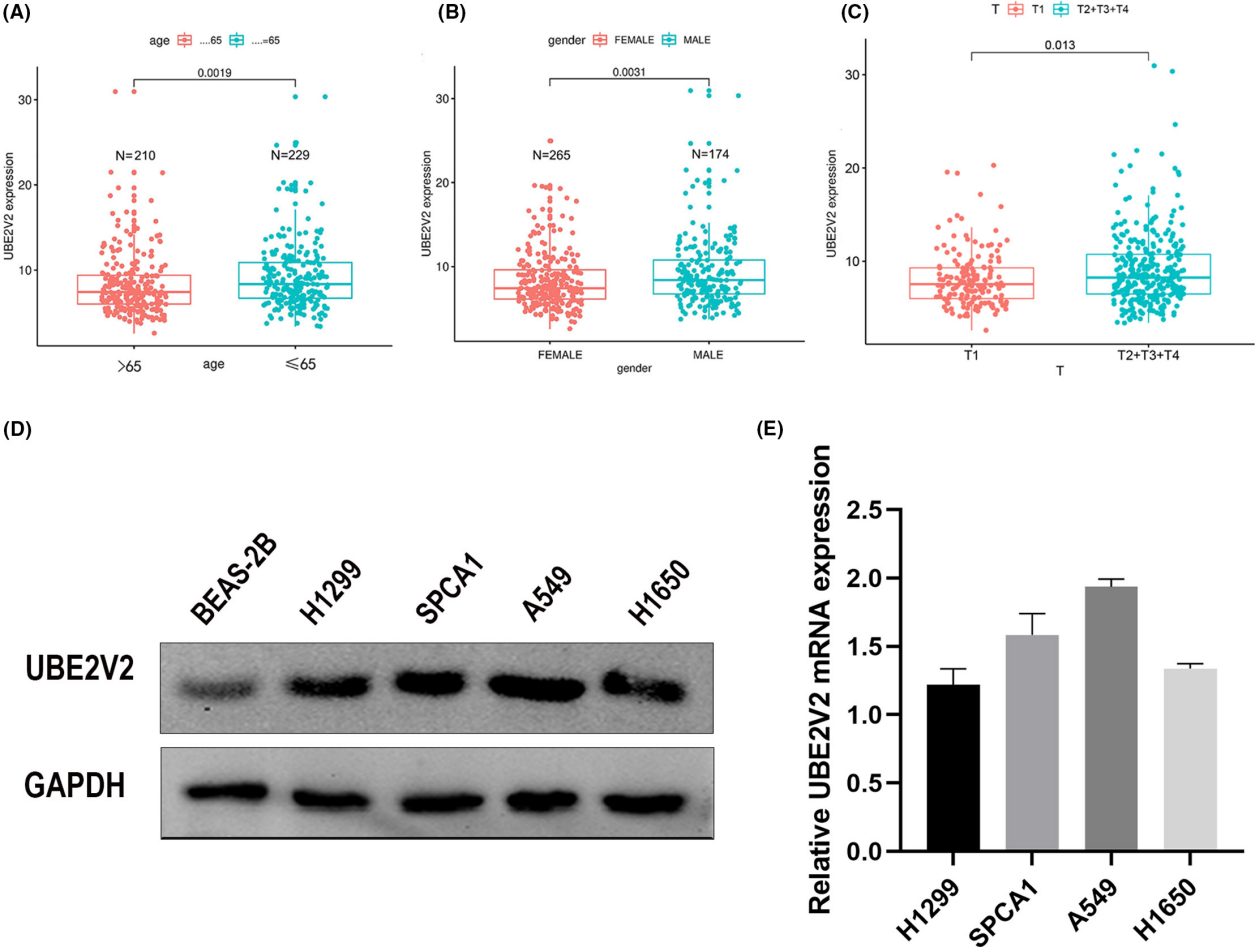
The correlation between UBE2V2 and clinical parameters, and its expression level in LUAD cells. (A) age (*p* = 0.0019), (B) gender (*p* = 0.0031), (C) tumor stage (T2 + T3 + T4 vs. T1 *p* = 0.013), (D) Expression of UBE2V2 in four LUAD cells and human small airway epithelial cells (BEAS‐2B) measured by Western blotting. (E) The relative mRNA expression in the four LUAD cells based on PCR. (UBE2V2, ubiquitin‐conjugating E2 enzyme variants 2; TCGA, The Cancer Genome Atlas). Multiple comparisons were analyzed using ANOVA with posttest. Two groups were analyzed using unpaired *t*‐test. PCR, polymerase chain reaction.

To explore the relationship between UBE2V2 and the clinicopathological parameters of LUAD patients. We first used the “ggplot2” package to explore whether UBE2V2 was obviously associated with Gender (*p* = 0.0031), age (*p* = 0.0019), and T classification (*p* = 0.013) based on TCGA (Figure 3A–C). Then, we used IHC to analyze the expression of UBE2V2 in 91 samples of LUAD patients and its relationship with clinicopathological parameters. Among them, 41 samples (45.05%) in total showed a low expression level of UBE2V2, and the other 50 (54.95%) samples showed a high expression level of UBE2V2 (Table 1). Collectively, the results of IHC showed that UBE2V2 was correlated with three factors: Gender (*p* = 0.043), tumor stage (*p* = 0.042), and lymph node metastasis (*p* = 0.002) in LUAD (Table 1).

### 3.4 GSEA identified UBE2V2‐related signaling pathways

**FIGURE 4:**
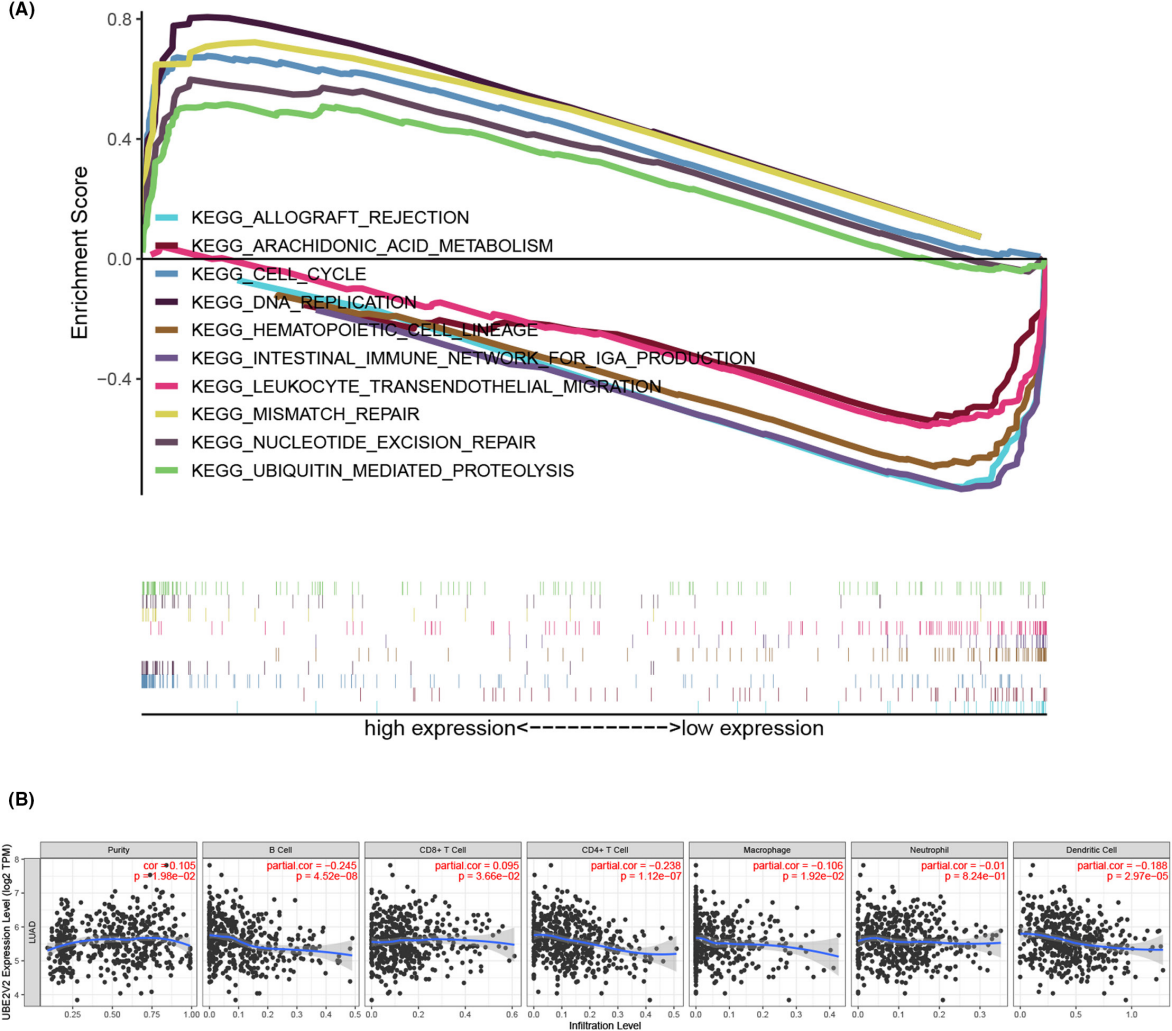
KEGG signaling pathway and tumor infiltrating lymphocytes. (A) The important related genes and KEGG signal pathway related to UBE2V2 were obtained in LUAD through GSEA, which included five positive and five negative correlation pathways, respectively. (B) Correlations between expression of UBE2V2 and tumor infiltrating lymphocytes (TILs) in LUAD based on the TIMER. UBE2V2 correlated with abundance of tumor purity (*r* = 0.105, *p* = 0.019) and CD8^+^ T cells (*r* = 0.095, *p* = 0.036), B cells (*r* = −0.245, *p* < 0.0001), CD4^+^ T cells (*r* = −0.238, *p* < 0.0001), macrophages (*r* = −0.106, *p* = 0.019), and dendritic cells (*r* = −0.188, *p* < 0.0001). (UBE2V2, ubiquitin‐conjugating E2 enzyme variants 2; KEGG, Kyoto Encyclopedia of Genes and Genomes; GSEA, gene set enrichment analysis; LUAD, lung adenocarcinoma).

The signaling pathway regulated by UBE2V2 in the development of LUAD was obtained by GSEA. GSEA showed remarkable differences (*p* value <0.050, FDR <0.050) compared to enrichment of KEGG pathways in samples with high UBE2V2 expression. Based on the normalized enrichment score (NES), the enriched signaling pathway with the most significant phenotypic enrichment was selected. KEGG pathway analysis identified five pathways that had a positive correlation with UBE2V2 expression: DNA replication, cell cycle, mismatch repair, ubiquitin‐mediated proteolysis, and nucleotide excision repair. The five pathways with the strongest negative correlation were arachidonic acid metabolism, allograft rejection, hematopoietic cell lineage, leukocyte transendothelial migration, and intestinal immune network for IgA production, as shown in Figure 4A. These results indicated that the pathways regulating DNA damage repair and the immune microenvironment are critically important in LUAD patients.

### 3.5 The association between UBE2V2 and tumor infiltrating lymphocytes (TILs) in LUAD

The association between UBE2V2 expression and the level of infiltration of different immune cells was studied through the “Gene” panel of TIMER (Figure 4B). The results revealed that UBE2V2 was positively related to tumor purity in LUAD (*r* = 0.105, *p* = 0.019) and CD8^+^ T cells (*r* = 0.095, *p* = 0.036) but negatively related to B cells (*r* = −0.245, *p* < 0.0001), CD4^+^ T cells (*r* = −0.238, *p* < 0.0001), macrophages (*r* = −0.106, *p* = 0.019), and dendritic cells (*r* = −0.188, *p* < 0.0001). Co expression analysis was used to count the genes co‐expressed UBE2V2 in LUAD. The 10 genes with the highest correlation coefficients were made into circle chart (Figure S1A and table S1). The correlation analysis between UBE2V2 and COPS5 (R=0.67,P < 0.001) was shown in figure S1B.

### 3.6 Expression of UBE2V2 in four LUAD cell lines

**FIGURE 5:**
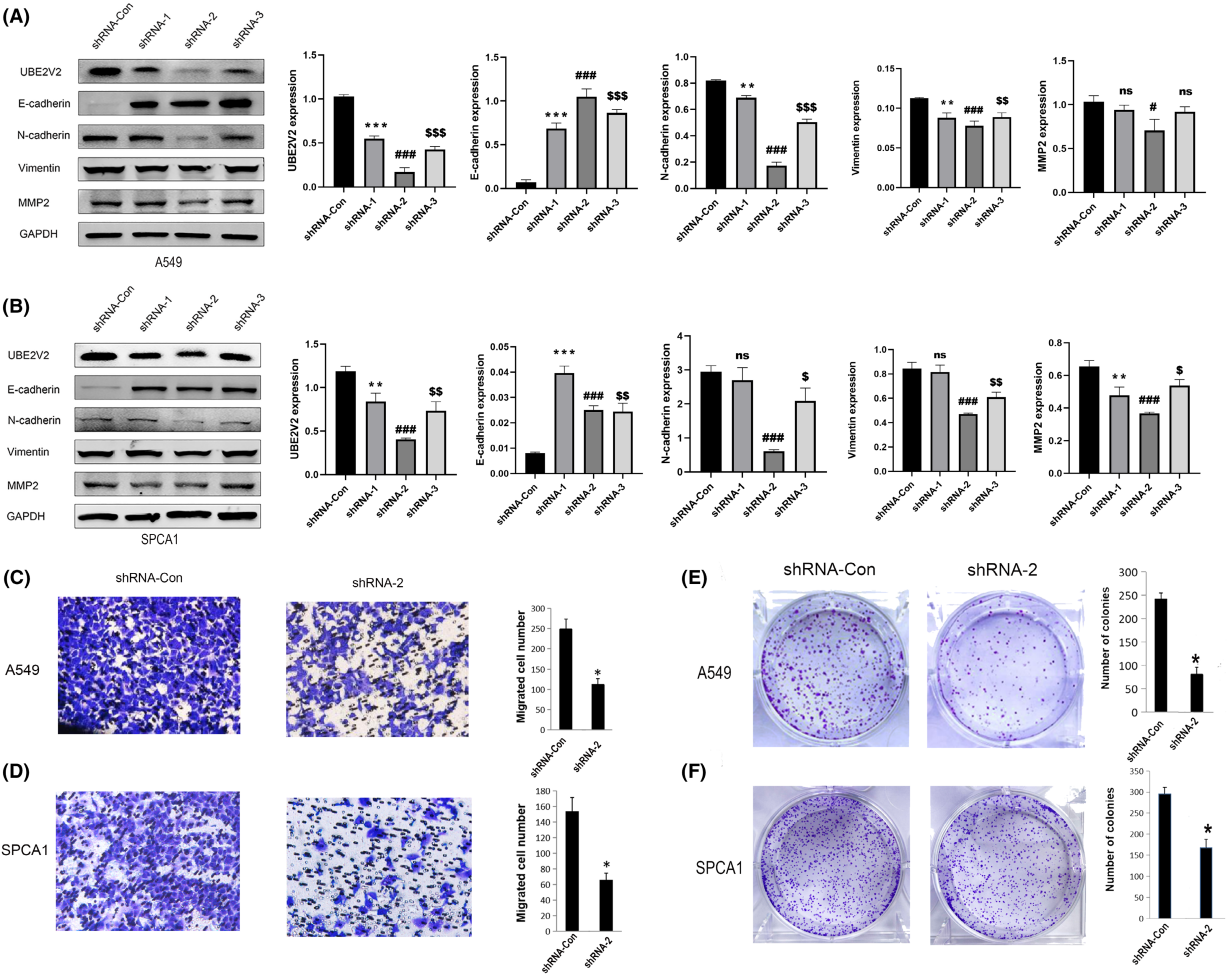
Transwell assay and the relationship between UBE2V2 and EMT‐related markers in LUAD cell lines. (A, B) Western blotting detected the knockdown efficiency of UBE2V2 (shRNA‐1,2,3) compared to the control group (shRNA‐Con) in A549 and SPCA1 cells, and the expression changes of EMT‐related proteins (E‐cadherin, N‐cadherin, Vimentin, and MMP2) after knockdown of UBE2V2. (C, D) Transwell assay showed that the knockdown UBE2V2 (shRNA‐2) significantly reduced the number of migrating cells than the control group (shRNA‐Con) in A549 and SPCA1 cells. (E, F) Knockdown of UBE2V2 (shRNA‐2) reduced the number of cell clones compared to control group (shRNA‐Con) in A549 and SPCA1 cells by colony formation assays. The data (mean ± standard deviation) between two groups were compared using unpaired *t*‐test. *p < 0.05, **p < 0.01, ***p < 0.001 (shRNA‐1 vs. shRNA‐Con); ^#^ *p* < 0.05, ^###^ *p* < 0.001 (shRNA‐2 vs. shRNA‐Con); ^$$^ *p* < 0.01, ^$$$^ *p* < 0.001 (shRNA‐3 vs shRNA‐Con); ^ns^ *p* > 0.05. (UBE2V2, ubiquitin‐conjugating E2 enzyme variants 2; EMT, epithelial–mesenchymal transition; LUAD, lung adenocarcinoma; sh‐, short hairpin; Con, negative control).

For further investigating the molecular mechanism underlying UBE2V2 in regulating LUAD, two LUAD cell lines were selected from four LUAD cell lines for application in the next experiment. The expression of UBE2V2 in four kinds of lung adenocarcinoma cells was higher than that in normal bronchial epithelial cells (Figure 3D). The results of Western blotting showed that the protein and mRNA expression levels of UBE2V2 in A549 and SPCA1 cells were relatively higher than in H1299 and H1650 cells (Figure 3D,E). Therefore, A549 and SPCA1 cells were selected for next study. Then, UBE2V2‐shRNAs or Con‐shRNA lentiviruses were used to infect A549 and SPCA1 cells to specifically interfere the expression of UBE2V2. The results of WB showed that the expression of UBE2V2 was significantly downregulated by UBE2V2‐shRNAs lentiviruses infection compared to shRNA‐Con lentiviruses infection (Figure 5A, B).

### 3.7 Knockdown of UBE2V2 suppressed the migration of A549 and SPCA1 cells

We applied Transwell analysis to investigate whether knockdown of UBE2V2 affected the migration ability of LUAD cells. The results demonstrated that knockdown of UBE2V2 (shRNA‐2) remarkably inhibited the migration ability of A549 and SPCA1 cells (Figure 5C, D). Thus, the results of Transwell analysis indicated that UBE2V2 might be related to the migration of LUAD cells.

### 3.8 Knockdown of UBE2V2 inhibited the EMT process in SPCA1 and A549 cells

Tumor metastasis involves many replication processes, including the loss of polarity on the top‐bottom surface of epithelial cells, the absence of tight junctions and adhesion junctions between cells and the degradation of extracellular matrix and basement membrane. To investigate whether UBE2V2 was related to epithelial–mesenchymal transition (EMT) in LUAD cells, EMT‐related proteins, including E‐cadherin, N‐cadherin, vimentin, and MMP2, were studied via Western blotting. The results showed that the expression of a cellular epithelial protein (E‐cadherin) significantly increased, while the expression of proteins characteristic of mesenchymal cells, such as vimentin, N‐cadherin, and MMP2, was obviously reduced after knockdown of UBE2V2 (shRNA‐2) in A549 and SPCA1 cells (Figure 5A, B).

### 3.9 Knockdown of UBE2V2 suppressed the proliferation of A549 and SPCA1 cells

**FIGURE 6:**
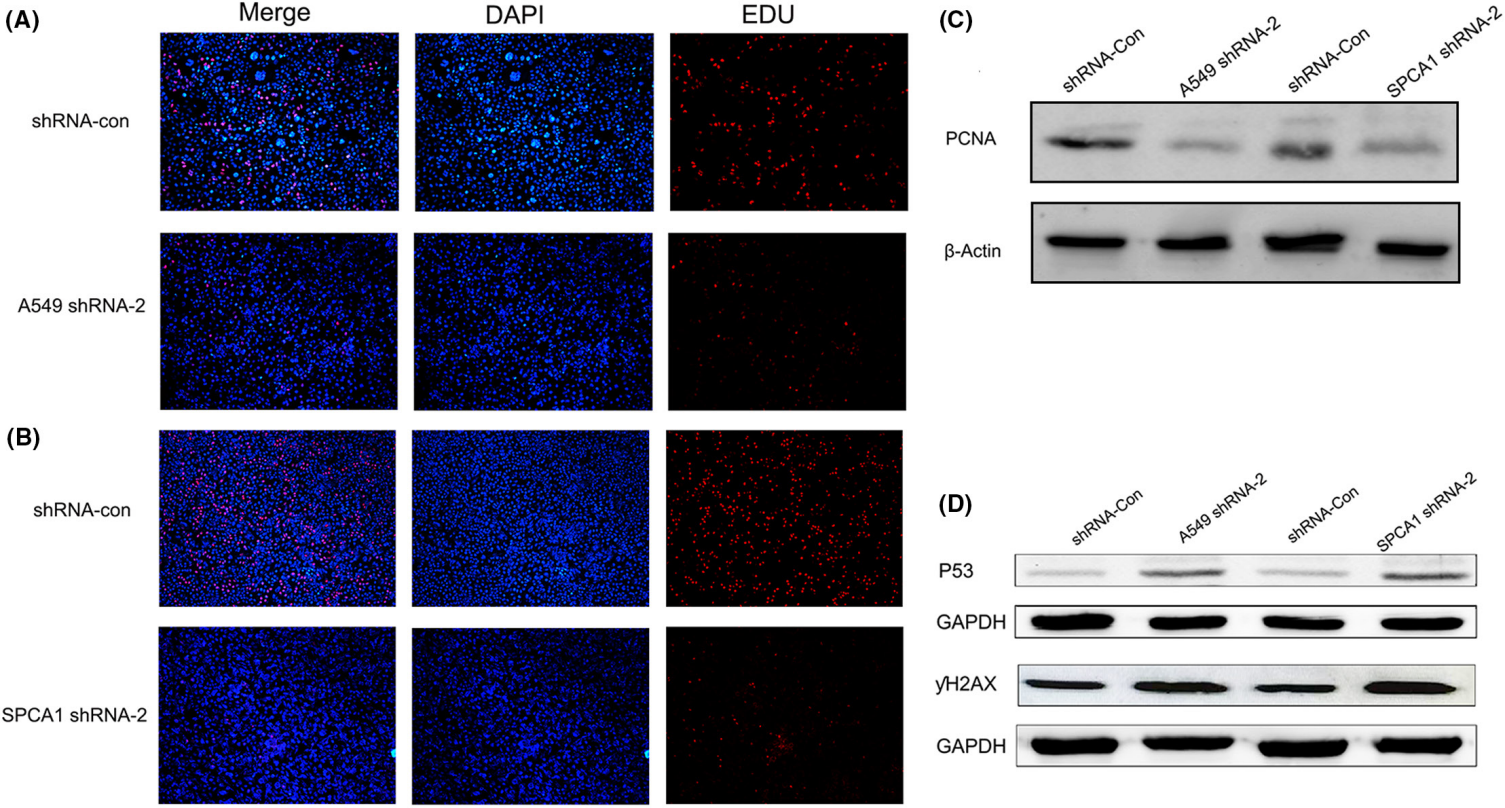
UBE2V2 promoted LUAD cells proliferation. (A, B) EDU assay confirmed that the proliferation ability of A549 cell lines and SPCA1 cell lines decreased after knockdown of UBE2V2. (C) Knockdown of UBE2V2 (shRNA‐2) obviously reduced the expression of proliferating cell nuclear antigen (PCNA) in A549 and SPCA1 cells. (D) Knockdown of UBE2V2 upregulated the expression of P53 and ƴH2AX in A549 and SPCA1 cells. (UBE2V2, ubiquitin‐conjugating E2 enzyme variants 2; LUAD, lung adenocarcinoma; sh‐, short hairpin; Con‐, negative control).

To explore whether UBE2V2 affected the proliferation of LUAD, a series of experiments was performed. Colony formation assays showed that the number of colony cells with UBE2V2 knockdown (shRNA‐2) was clearly reduced compared to shCon cells (Figure 5E, F). Meanwhile, EDU assay confirmed that the proliferation ability of A549 cell line and SPCA1 cell line decreased after knockdown of UBE2V2 (shRNA‐2), respectively (Figure 6A, B). WB analysis showed that the proliferation marker PCNA was also remarkably reduced in A549 and SPCA1 cells after knockdown of UBE2V2(shRNA‐2) (Figure 6C).

### 3.10 Knockdown of UBE2V2‐induced apoptosis and led to cell cycle arrest in A549 and SPCA1 cells

**FIGURE 7:**
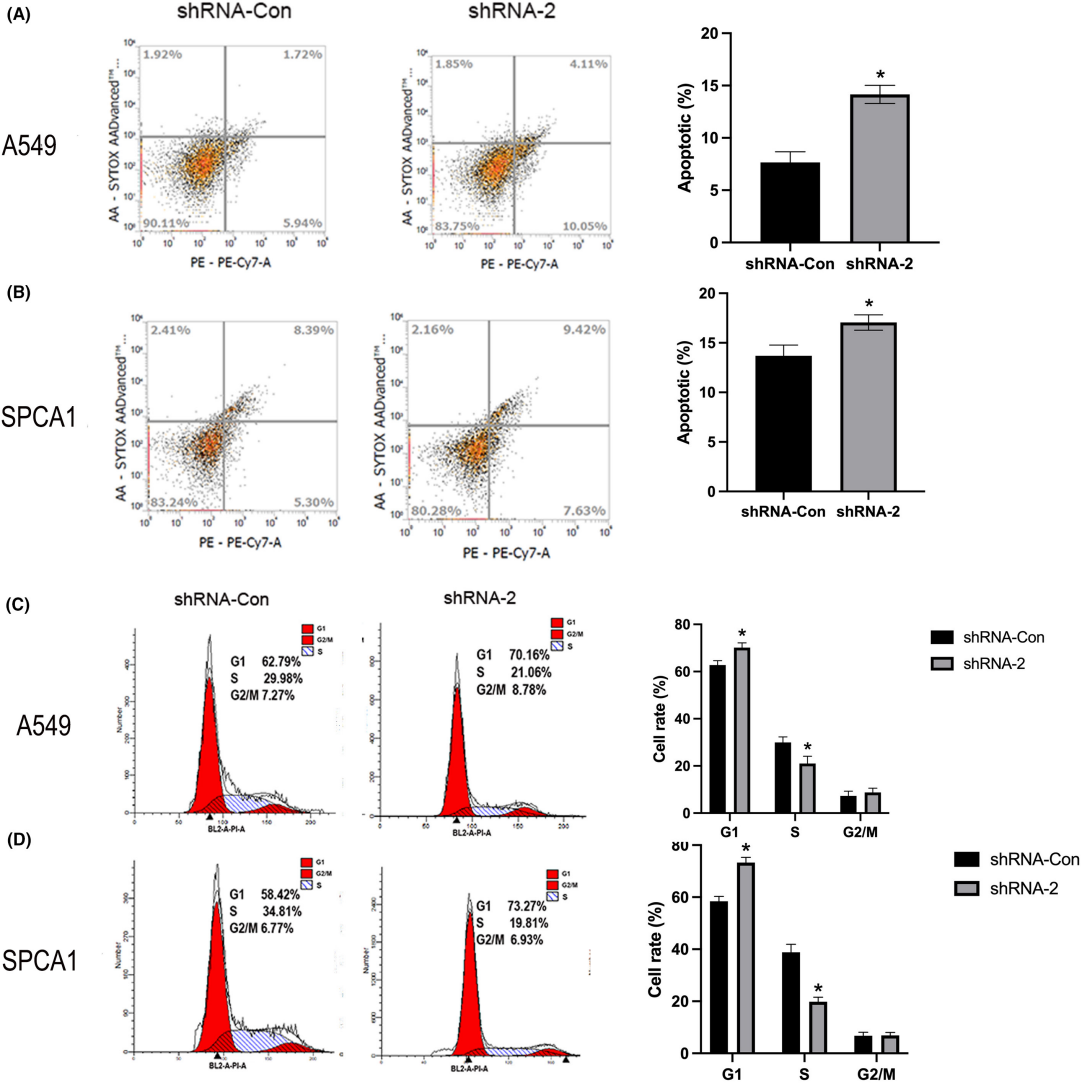
Knockdown of UBE2V2 induced apoptosis and led to cell cycle arrest in LUAD cells. (A, B) Knockdown of UBE2V2 (shRNA‐2) significantly increased the number of apoptotic cells compared to control group (shRNA‐Con) in A549 and SPCA1 cells. (C, D) Knockdown of UBE2V2 (shRNA‐2) increased the proportion of cells in the GI phase and remarkably diminished the proportion of cells in the DNA synthesis phase (S phase). The data (mean ± standard deviation) between two groups were compared using unpaired *t*‐test. *p < 0.05.(UBE2V2, ubiquitin‐conjugating E2 enzyme variants 2; LUAD, lung adenocarcinoma; sh‐, short hairpin; Con‐, negative control).

Through flow cytometry analysis, we detected the proportion of healthy cells, early/late apoptotic cells, and necrotic cells in UBE2V2 knockdown cells. We observed a remarkable increase in the percentage of early/late apoptotic cells in UBE2V2‐knockdown cells compared to shCon‐cells (Figure 7A,B). Flow cytometry was further used to analyze whether knockdown of UBE2V2 affects the cell cycle in LUAD cells. After knockdown of UBE2V2 (shRNA‐2), cell replication stagnated in the early stage of DNA synthesis (G1 phase): the proportion in the G1 phase increased significantly, yet the proportion was remarkably diminished in the DNA synthesis phase (S phase) (Figure 7C,D). Moreover, the results of WB showed that knockdown of UBE2V2 (shRNA‐2) upregulated the expression of P53 and ƴH2AX in SPCA1 and A549 cells, respectively (Figure 6D).

## 4 DISCUSSION

UBE2V2, a ubiquitin‐conjugating enzyme variant protein, belongs to a subfamily of E2 protein families.^17^ UBE2V2 lacking the active site of cysteine binds to specific UBC13 to form a heterodimer, which catalyzes ubiquitin to arrange a K63‐linked noncanonical polyubiquitin chain.^7^, ^8^, ^18^ The special structural features of UBE2V2 may result in different functions in cancer from other classic E2 enzymes. In previous studies, UBE2V2 was highly expressed in LUAD, and IHC confirmed that UBE2V2 may be an independent prognostic factor in patients with LUAD. However, whether UBE2V2 affects the progression of LUAD cells has not been studied in vitro.

In this report, we also confirmed that UBE2V2 was highly expressed in LUAD, and UBE2V2 could serve as an independent prognostic factor for LUAD patients based on the TCGA, GEO, and IHC. These results are consistent with previous studies, which strengthen our belief that UBE2V2 may be involved in the development of LUAD cells. Then, we further explored that the mRNA and protein expression levels of UBE2V2 in LUAD samples were significantly higher than in normal samples based on WB and RT–qPCR. Low UBE2V2 expression was associated with a better survival prognosis than high UBE2V2 expression in LUAD, which was forecasted by the TCGA and GEO. The same Kaplan–Meier survival curve result was verified via IHC. Multivariate Cox analyses of both IHC and bioinformatics indicated that UBE2V2 expression was a potential independent marker of poor prognosis in LUAD.

The results of IHC analysis suggested that UBE2V2 was related to lymph node metastasis. Based on the IHC results, we tested whether UBV2V2 at the cellular level was related to the migration of LUAD cells. Transwell assays showed that knockdown of UBE2V2 inhibited the migration ability of LUAD cells. This result was consistent with IHC, which indicated that UBE2V2 might affect the invasion and migration ability of LUAD cells. However, there is no relevant research to explain the mechanism by which UBE2V2 increases the invasion and metastasis ability of tumors. Inhibiting the expression of UBE2V2 upregulated the expression of E‐cadherin (an EMT marker) only in melanoma.^12^ Therefore, we speculated that UBE2V2 also promotes the metastasis of LUAD by regulating EMT‐related proteins. Our data indicated that knockdown of UBE2V2 increased the expression of E‐cadherin while decreasing the expression of vimentin, N‐cadherin, and MMP2. The above experimental results might reveal that knockdown of UBE2V2 inhibited metastasis of LUAD cells by regulating EMT‐related proteins. The specific mechanism by which UBE2V2 causes EMT in LUAD cells needs further study.

Moreover, UBE2V2, as the first to be discovered, plays an important role in DNA damage repair in eukaryotes.^9^ In organisms, DNA is extremely susceptible to damage by various external and internal factors. The DNA damage repair system is highly conserved from yeast to humans, including nuclear excision repair, base excision repair, photoresurrection, mismatch repair, double‐strand break repair, and cross‐damage replication.^19^ UBC13‐UBE2V2 interacted with the DNA binding protein RAD5 (an E3 ligase) and was recruited to chromatin.^20^, ^21^ The polyubiquitin chain formed by the RAD5‐UBE2V2‐UBC13 complex in chromatin mediates recombination repair after DNA replication by an error‐free template conversion.^22^ In this study, KEGG pathway analysis predicted that UBE2V2 was mainly positively related to the cell cycle, nucleotide excision repair, mismatch repair, and DNA replication. Bioinformatics suggested that UBE2V2 might be related to cell proliferation and apoptosis. Proliferating cell nuclear antigen (PCNA) is closely related to cell DNA synthesis and plays an important role in the initiation of the proliferation of various tumor cells.^19^, ^23^ PCNA changes dynamically with the cell cycle.^24^ It shows no obvious expression in the G0 ~ G1 phase, and its expression increases to a peak in the S phase and decreases significantly in the G2 ~ M phase. The change in PCNA expression is consistent with DNA synthesis and reflects the state of cell proliferation.^25^ Our results showed that knockdown of UBE2V2 downregulated the expression of PCNA in SPCA1 and A549 cells by WB. A remarkable decrease in the percentage of S phase and a tremendous improvement in the percentage of G1 phase were observed after knockdown of UBE2V2 in LUAD cells. Therefore, we speculated that UBE2V2 in lung cancer increased PCNA activity in the S phase, which would induce the transfer of damaged DNA from the replication mechanism to the trans‐injury synthesis pathway mediated by the RAD5‐UBE2V2‐UBC13 protein and eventually lead to lung cancer cells developing resistance to a variety of chemotherapeutic drugs.

P53, as a tumor suppressor protein, regulates cell proliferation and apoptosis in organisms.^26^ During DNA damage, the expression of P53 showed an increasing trend.^27^ The ubiquitin proteasome system plays an important role in the proteolysis of P53, which occurs through the UBC13‐mediated polyubiquitin chain.^28^ UBC13 is linked to p53 and increases the cytoplasmic pool of monomeric p53 to restrain the formation of nuclear tetramerization of p53.^29^ This study showed that knockdown of UBE2V2 upregulated the expression of P53 in SPCA1 and A549 cells by WB. The above results indicate that UBC13 and UBE2V2 form a specific heterodimer to mediate lysine 63‐specific protein ubiquitination and then bind to the P53 protein to degrade it, thereby inhibiting the apoptotic ability of lung adenocarcinoma cells. In addition, our results found that knockdown of UBE2V2 induced the formation of ƳH2AX. This might indicate that the arrest of G1 phase after knocking down UBE2V2 was related to increased DNA damage.

The immune infiltration of tumors has attracted attention in recent years. The tumor microenvironment (TME) is one of the important factors leading to the malignant progression of tumors.^30^ It has been found in many tumor models that CD + 4 and CD + 8 T cells inhibit tumor growth and cause the regression of solid tumors.^31^, ^32^, ^33^ CD4+ T cells cause tumor regression through an IFN‐c‐dependent mechanism, which occurs by recruiting macrophages and/or eosinophils.^34^ We used the TIMER database to reveal the link between UBE2V2 and different types of immune cell infiltration levels in LUAD. Our data revealed that UBE2V2 was significantly negatively associated with CD4+ T cells, macrophages, and B cells, and positively correlated with CD8 + T cells. This suggests that overexpression of UBE2V2 inhibits the infiltration of immune cells in LUAD, which may also provide us with a new idea for immunotherapy.

## 5 CONCLUSIONS

In summary, we predicted the expression pattern of UBE2V2 in LUAD through predictive analysis based on the TCGA database and verified the prediction results through a series of in vitro cell experiments. Our data indicated that UBE2V2 was overexpressed in LUAD and could be used as an independent prognostic indicator of LUAD. Second, we studied the function and mechanism of UBE2V2 in regulating LUAD through in vitro experiments. The results showed that UBE2V2 caused LUAD cell metastasis by activating EMT‐related proteins. Our results also revealed that UBE2V2 could increase the proliferation capacity and reduce the apoptosis of LUAD cells. However, our experiments were limited to TCGA predictions and in vitro studies. Although we tried to expand the sample size of the experimental data, the results are inevitably affected by sample size and selection bias. At the same time, the knockdown efficiency is not obvious in some cell lines. We will use another shRNA to knockdown UBE2V2 (similar or higher efficiency than shRNA‐2) and validate their results. We will also try to establish a UBE2V2‐deletion mouse model to further analyze the effects of UBE2V2 on the metastasis, proliferation, and apoptosis of LUAD to improve the reliability of the experiment. This will exert a profound impact on the further application of our experimental results in clinical practice.

## AUTHOR CONTRIBUTIONS

**Zheng Yang:** Data curation (equal); software (equal); writing – original draft (equal). **Gujie Wu:** Software (equal). **Jianmei Zhao:** Investigation (equal). **Guanglin Shi:** Software (equal). **Juan Zhou:** Writing – review and editing (equal). **Xiaoyu Zhou:** Writing – review and editing (equal).

## FUNDING INFORMATION

This work was supported by Nantong Science and technology plan (No. MS22019001; HS2018003) and Nantong Science and Technology Plan (No. JCZ18109).

## CONFLICT OF INTEREST STATEMENT

The authors declare that they have no competing interests.

## ETHICS STATEMENT

This study was officially recognized for it strictly carried out the procedures for care and use admitted by the Ethics Committee of the Affiliated Hospital of Nantong University (Ethic Number: 2018‐K020). Written informed consents were obtained from all participants.

## Supporting information


Figure S1

Table S1

## Data Availability

The datasets used and/or analyzed during the current study are available from the corresponding author upon reasonable request.
