# Supplementary figures and images for: UBE2V2 promotes metastasis by regulating EMT and predicts a poor prognosis in lung adenocarcinoma
Source: Cancer Med. 2023 Sep 27;12(19):19850–65. doi: 10.1002/cam4.6566 (PMC10587983; doi:10.1002/cam4.6566)

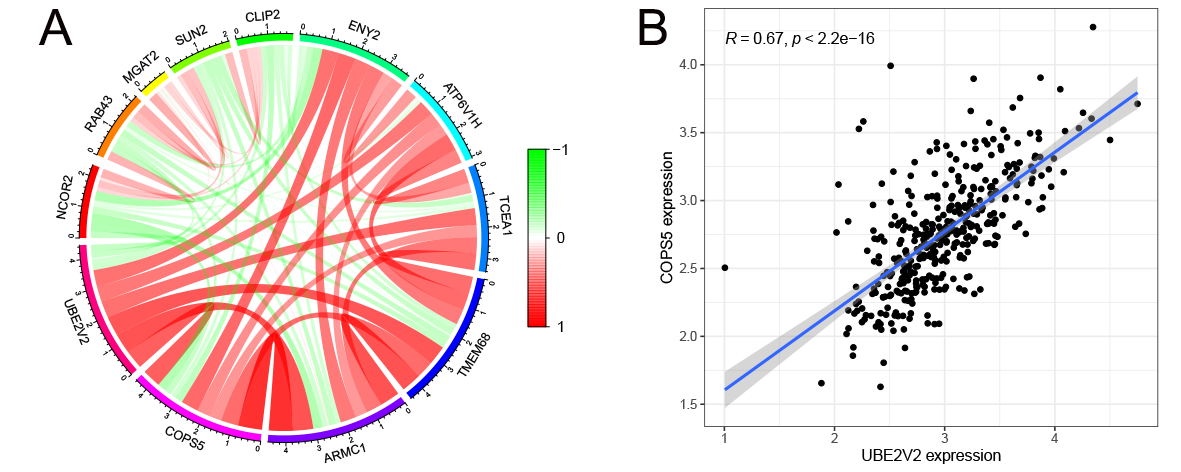

Supplement: Supplementary file 1 — Figure S1 [file CAM4-12-19850-s002.tif]
