# Supplementary material for: UBE2V2 promotes metastasis by regulating EMT and predicts a poor prognosis in lung adenocarcinoma
Source: Cancer Med. 2023 Sep 27;12(19):19850–65. doi: 10.1002/cam4.6566 (PMC10587983; doi:10.1002/cam4.6566)
Supplement: Supplementary file 2 — Table S1 [file CAM4-12-19850-s001.docx]

**Figure S1 Co-expression analysis of UBE2V2 in LUAD.**

(A)The 10 genes with the highest correlation coefficients were made into a circle chart(P<0.05) (Red means positive correlation, green means negative correlation). (B) Correlation analysis of UBE2V2 and COPS5 (R=0.67 P<0.001). Pearson test was used to count the genes co-expressed with UBE2V2 in LUAD.

Table S1 **Co-expression analysis of UBE2V2 in LUAD.**

| Query | Gene | cor | pvalue |
| --- | --- | --- | --- |
| UBE2V2 | NCOR2 | -0.41989 | 1.90E-17 |
| UBE2V2 | RAB43 | -0.41383 | 6.01E-17 |
| UBE2V2 | MGAT2 | -0.40256 | 4.84E-16 |
| UBE2V2 | SUN2 | -0.4003 | 7.28E-16 |
| UBE2V2 | CLIP2 | -0.38411 | 1.25E-14 |
| UBE2V2 | COPS5 | 0.672067 | 1.31E-50 |
| UBE2V2 | ARMC1 | 0.651774 | 1.01E-46 |
| UBE2V2 | TMEM68 | 0.647417 | 6.33E-46 |
| UBE2V2 | TCEA1 | 0.644687 | 1.96E-45 |
| UBE2V2 | ATP6V1H | 0.629126 | 1.01E-42 |
